# Optical coherence tomography angiography in eyes with good visual acuity recovery after treatment for optic neuritis

**DOI:** 10.1371/journal.pone.0172168

**Published:** 2017-02-13

**Authors:** Tomoaki Higashiyama, Yasuhiro Nishida, Masahito Ohji

**Affiliations:** 1 Department of Ophthalmology, Shiga University of Medical Science, Otsu, Japan; 2 Clinical Medical Education Center, Shiga University of Medical Science, Otsu, Japan; Justus Liebig Universitat Giessen, GERMANY

## Abstract

**Objective:**

To evaluate the retinal perfusion using optical coherence tomography (OCT) angiography in eyes with good visual acuity recovery after treatment for optic neuritis (ON).

**Methods:**

Seven eyes of seven patients with good visual acuity recovery after treatment for monocular ON and seven eyes of each fellow eye used as controls were studied. Retinal perfusion around the disc and at the macula was evaluated using OCT angiography. The retinal nerve fiber layer thickness was measured around the disc. The ganglion cell layer complex thickness or the ganglion cell layer plus the inner plexiform layer thickness were measured at the macula.

**Results:**

The retinal perfusions in all eyes with ON decreased around the disc and at the macula compared with those of the fellow eyes, as shown by OCT angiography (disc, *P* = 0.003; macula, *P* = 0.001). The retinal thicknesses in all eyes with ON also decreased around the disc and at the macula compared with those of the fellow eyes (disc, *P* < 0.001; macula, *P* = 0.003).

**Conclusions:**

Optic neuritis may cause not only retinal structural damage but also decreased retinal perfusion, even after the visual acuity recovered well after treatment.

## Introduction

Optic neuritis (ON) is an inflammation of the optic nerve that can be subclassified into various types, including multiple sclerosis (MS)-associated ON, neuromyelitis optica-associated ON, ON associated with systemic disorders such as infective conditions, and other idiopathic forms of ON without systemic disease such as solitary isolated ON [1]. In the acute phase of ON, inflammatory demyelination occurs, causing a conduction block and visual loss [1–3]. Visual acuity may still recover well following resolution of inflammation in most patients, even if the patients experience severe visual loss due to ON [1, 4].

However, the retinal structural damages may remain after resolution of inflammation because the axons are irreversibly lost due to ON, even if the visual acuity recovered after treatment. Previous studies reported that optical coherence tomography (OCT) showed that the retinal nerve fiber layer (RNFL) thickness around the disc and ganglion cell layer (GCL) thickness at the macula were decreased after ON [5–9]. The RNFL thickness around the disc and the GCL thickness at the macula decreased even in eyes with good visual acuity recovery after treatment for ON [8]. However, whether the retinal perfusion decreases in eyes with well recovered visual acuity after treatment for ON has not been reported.

OCT angiography, which was recently developed, can provide visualization of the retinal vascular flow. OCT angiography can also noninvasively evaluate retinal perfusion, including those of the capillaries [10–14], and can evaluate retinal perfusion in both the macula and disc [10–18]. We therefore evaluated the retinal perfusion around the disc and at the macula using OCT angiography in eyes with good visual acuity after treatment for ON.

## Methods

### Subjects

Seven eyes of seven patients (four female and three male patients) with good visual acuity recovery after treatment for ON were included in this observational cross-sectional study at the Department of Ophthalmology, Shiga University of Medical Science Hospital, from October 2015 to September 2016. This study was approved by the Institutional Review Board of Shiga University of Medical Science, and was conducted in accordance with the tenets of the Declaration of Helsinki. Written informed consent/assent was obtained from each patient in the study. When the patient enrolled in this study was minor, written informed consent and assent were obtained from each parent and patient. All patients visited our hospital soon after they suffered visual impairment. Magnetic resonance imaging (MRI) examinations were performed before treatment and monocular ON was diagnosed in all patients. Just after diagnosis of monocular ON, all patients were treated with methylprednisolone pulse therapy (all cases except Case 2 and 7, a daily dose of 1,000 mg on just 3 successive days; Case 2, a daily dose of 30 mg/kg on 3 successive days; Case 7, a daily dose of 1,000 mg on 3 successive days, weekly for 2 weeks). Patients were included in this study if the best-corrected visual acuities (BCVAs) of both eyes were 20/20 or better. There was no criterion of age or race in this study. All patients also underwent ophthalmological examinations, including measurements of BCVA, critical flicker frequency (CFF), and OCT.

### Retinal perfusion

The OCT angiography centered around the disc and at the macula were obtained using the RTVue XR Avanti (Optovue, Fremont, CA, USA), or the Cirrus HD-OCT 5000 (Carl Zeiss Meditec Inc, Dublin, CA, USA). The OCT angiograms were obtained after treatment. Using the RTVue XR Avanti, OCT angiograms centered around the disc (4.5 × 4.5 mm) and at the macula (6.0 × 6.0 mm) were obtained. The radial peripapillary capillary images were taken around the disc, and the superficial images were taken at the macula. The radial peripapillary capillary image was segmented with an inner boundary at the internal limiting membrane (ILM) and an outer boundary was set at 100 μm beneath the ILM. The superficial image was segmented with an inner boundary at 3 μm beneath the ILM and the outer boundary set at 15 μm beneath the inner plexiform layer (IPL). Using the Cirrus HD-OCT 5000, OCT angiograms centered around the disc (6.0 × 6.0 mm) and at the macula (6.0 × 6.0 mm) were obtained. The superficial images were taken around the disc and at the macula, and segmented between the ILM and the IPL.

Retinal perfusion in the eye with ON was compared with that in the fellow eye of each patient. The OCT angiograms were binarized using Image J software (National Institutes of Health, Bethesda, MD, USA; http://rsbweb.nih.gov/ij/index.html) to quantify the microvasculature [19]. The vessel density was defined as the percentage area occupied by the vessels in the image. The measurements included the characters “angio FLOW” in each image using the RTVue XR Avanti. The binarized setting, which set the vessel density of the control eye as approximately 50%, was used in both eyes of each patient.

### Retinal thickness

In cases using the RTVue XR Avanti, the RNFL thickness around the disc and ganglion cell layer complex (GCC) thickness at the macula were measured. GCC thickness was defined as the distance between the ILM and the IPL. In the cases using the Cirrus HD-OCT 5000, the RNFL thickness around the disc and the GCL + IPL thickness at the macula were measured. The average thickness was analyzed with the OCT software provided with each instrument.

### Statistical analysis

A paired *t*-test was used to compare the vessel densities and retinal thicknesses between the eyes with ON and the fellow eyes. SPSS statistical software for Windows, version 22 (IBM, Armonk, NY, USA) was used for all analyses. The data were expressed as the mean ± standard deviation (SD). *P* < 0.05 was considered statistically significant.

## Results

The mean age of the patients was 36.0 ± 20.3 years (mean ± standard deviation; range, 8–65 years). All patients had ON owing to acute isolated ON (Cases 1, 3, 5, and 6), MS (Cases 2, and 4) or anti-aquaporin-4 antibody-positive optic neuritis (Case 7). The mean follow-up period after treatments was 4.4 ± 2.2 months (range, 2–8 months). The characteristics of the patients are listed in Table 1. The OCT angiograms of three patients (Cases 1–3) were obtained using the RTVue XR Avanti and those were of four patients (Cases 4–7) were obtained using the Cirrus HD-OCT 5000. All patients had a BCVA of 20/20 or better. The mean CFF were 41.4 ± 5.9 Hz in the eye with ON and 43.1 ± 4.5 Hz in the fellow eye. The BCVA and CFF values of the patients are listed in Table 2.

**Table 1:** Patient characteristics.

Case	Age	Gender	Classification of optic neuritis	Eye with optic neuritis	Follow-up period after treatment (months)
1	14	F	Isolated optic neuritis	L	8
2	8	M	multiple sclerosis	R	2
3	27	M	Isolated optic neuritis	R	2
4	65	M	multiple sclerosis	L	5
5	46	F	Isolated optic neuritis	L	7
6	32	F	Isolated optic neuritis	R	3
7	60	F	Anti-aquaporin-4 antibody-positive optic neuritis	R	4

F, female; M, male; R, Right; L, Left

**Table 2:** The visual acuity and critical flicker frequency after treatment in cases 1–7.

Case	Best-corrected visual acuity		Critical flicker frequency	
	Eye with optic neuritis	Fellow eye	Eye with optic neuritis	Fellow eye
1	20/12	20/12	41	42
2	20/12	20/20	39	41
3	20/12	20/12	44	44
4	20/16	20/12	33	38
5	20/16	20/12	48	49
6	20/12	20/12	50	50
7	20/12	20/12	35	38

The mean binarized vessel density around the disc was 42.2 ± 3.9% in the eyes with ON and 50.6 ± 0.7% in the fellow eyes. Those at the macula were 41.9 ± 3.0% in the eyes with ON and 50.4 ± 1.3% in the fellow eyes (Table 3). The retinal perfusion around the disc and at the macula in all eyes with ON decreased compared with that of the fellow eyes (disc, *P* = 0.003; macula, *P* = 0.001). The mean retinal thickness around the disc was 75.6 ± 11.9 μm in the eyes with ON and 92.9 ± 10.2 μm in the fellow eyes. Those at the macula were 74.5 ± 10.1 μm in the eyes with ON and 87.1 ± 8.8 μm in the fellow eyes (Table 4). The retinal thickness around the disc and at the macula in all eyes with ON also decreased compared with those of the fellow eyes (disc, *P* < 0.001; macula, *P* = 0.003).

**Table 3:** Percentages of vessel densities around the disc and at the macula in cases 1–7.

Case	Disc		Macula	
	Eye with optic neuritis	Fellow eye	Eye with optic neuritis	Fellow eye
1	47.4	50.9	44.8	49.8
2	45.2	51.5	44.4	50.9
3	44.8	50.0	36.2	50.1
4	34.7	51.6	39.2	50.0
5	42.8	50.3	41.8	53.4
6	39.3	50.6	42.6	49.2
7	41.5	49.4	44.6	49.4

**Table 4:** Retinal thicknesses (μm) around the disc and at the macula in cases 1–7.

Case	Disc		Macula	
	Eye with optic neuritis	Fellow eye	Eye with optic neuritis	Fellow eye
1	71	93	86	92
2	64	75	72	79
3	92	101	91	101
4	56	80	79	95
5	81	99	63	83
6	88	100	67	74
7	77	102	64	86

**Fig 1:**
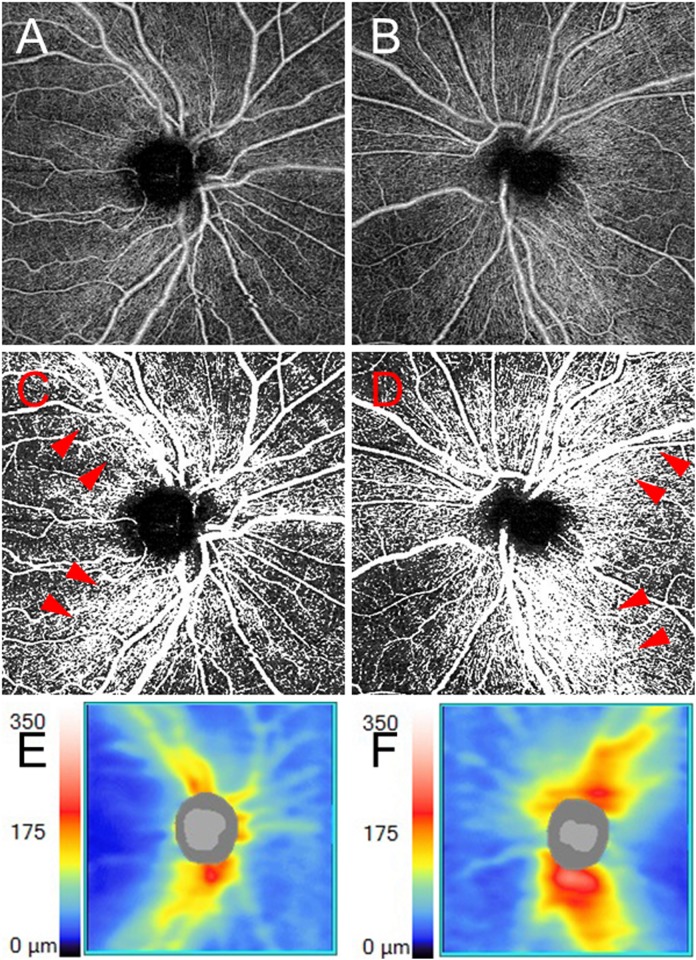
The optic disc findings in Case 7. **A, B** The optical coherence tomography (OCT) angiogram around the disc in the right eye (**A**) and the left eye (**B**). **C, D** The binarized images of the OCT angiograms around the disc of the right eye (**C**) and of the left eye (**D**). Decreased retinal perfusion is present in the right eye with optic neuritis. Notably, the retinal perfusion around arcade vessels (arrowheads) in the right eye decreased compared with that of the fellow eye. **E, F** Retinal nerve fiber layer (RNFL) thickness maps in the right eye (**E**) and the left eye (**F**). RNFL loss is present in the right eye.

**Fig 2:**
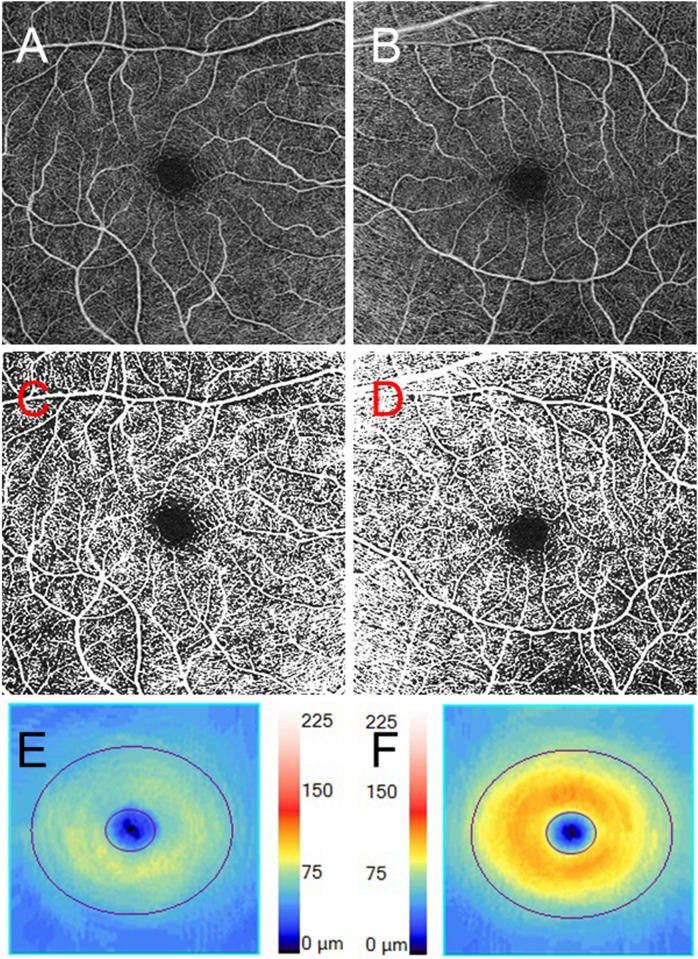
The macular findings of Case 7. **A, B** The optical coherence tomography (OCT) angiogram of the macula in the right eye (**A**) and the left eye (**B**). **C, D** The binarized images of the OCT angiograms of the macula of the right eye (**C**) and the left eye (**D**). Decreased retinal perfusion is present in the right eye with ON. **E, F** The ganglion cell layer + the inner plexiform layer (GCL+IPL) thickness map from the right eye (**E**) and the left eye (**F**). GCL+IPL loss is present in the right eye.

One representative case (Case 7) of a 60-year-old female is shown. The patient noticed right vision loss. Her BCVA was no light perception in the right eye and 20/12 in the left eye. The CFF value in the right eye was undetectable owing to no light perception while the value in the left eye was 34 Hz. A relative afferent pupillary defect was detected in the right eye. MRI results showed a high intensity in the right optic nerve. The anti-aquaporin-4 antibody result was positive. A diagnosis of anti-aquaporin-4 antibody-positive ON was made in the right eye and she was treated with methylprednisolone pulse therapy (a daily dose of 1,000 mg for 3 successive days weekly for 2 weeks). After the treatment, the BCVA recovered to 20/12 and the CFF value recovered to 35 Hz in the right eye. The retinal perfusions around the disc and at the macula in the eye with ON decreased compared with those of the fellow eye as determined by OCT angiography. The retinal thicknesses around the disc and at the macula in the eye with ON also decreased compared with those of the fellow eye. The OCT angiograms and the retinal thickness maps around the disc and at the macula in the patient are shown in Figs 1 and 2.

## Discussion

The current study clearly showed that the retinal perfusion around the disc and at the macula decreased as determine by OCT angiography in all eyes, even after good visual acuity recovery from ON. The retinal thicknesses around the disc and at the macula were also decreased in all eyes with ON. These results suggested that ON caused not only retinal structural damage, but also a decrease of retinal perfusion even after the visual acuity recovered well after treatment.

As determined by OCT angiography, the retinal perfusion decreased in eyes with good visual acuity recovery after treatment for ON. To the best of our knowledge, the retinal perfusion in eyes with good visual acuity recovery after ON has not been reported, while retinal thickness decreases have been reported previously [8]. In a study using OCT angiography for MS eyes, Wang et al. reported decreased retinal perfusion in eyes of MS patients with an ON history, whose visual acuity was at least 20/200 in either eye [16]. The report suggested possible causes for the decreased retinal perfusion, including MS damage, a reduced number of nerve fibers, and damages to the optic nerve head and the RNFL. The damage could reduce metabolic activities and the reduction of metabolic activity could lower retinal perfusion. In the present study, the retinal perfusion decreased in eyes with good visual acuity recovery after treatment. Therefore, although the visual acuity recovered well after treatment, the retinal perfusion could also decrease following a decrease of retinal thickness.

We previously reported the retinal perfusion results in the eyes of patients with other optic nerve diseases, including from those cases with non-arteritic anterior ischemic optic neuropathy and chiasmal compression [20, 21]. OCT angiography of the disc and macula showed decreased retinal perfusion in the retina, corresponding to the decreased retinal thickness reported in the study of non-arteritic anterior ischemic optic neuropathy [20]. A decrease in peripapillary retinal perfusion correlated with the quadrants of the visual field defects in the report of chiasmal compression [21]. The retinal perfusion results from the eyes with ON also decreased in our current study. Therefore, the retinal perfusions may decrease in the eyes after neuro-optical damage.

The present study has several limitations. First, we could not show changes of retinal perfusion before and after the treatment because the examinations were performed after treatment for ON. Second, it was sometimes difficult to clearly distinguish the slight decrease of retinal perfusion in the OCT angiograms. However, binarized vessel densities were used to quantitatively evaluate the angiograms in this study. Third, the durations between the examinations and the treatments were short. Costello et al. reported that thinning of the RNFL tended to occur within 3–6 months after ON [22]. Therefore, in this study the retinal structures and retinal perfusions might have decreased more. Fourth, the sample size was small. Only seven eyes of seven patients were enrolled in this study. However, consistent results were obtained in all seven cases. Fifth, the binarized vessel densities were not compared among the different patients because the binarized settings of the OCT angiograms were individually chosen. However, because the densities were individually measured using the same settings, the binarized vessel densities could be compared between both eyes of each patient. Sixth, Doppler angiography of the ophthalmic artery and carotid artery was not performed in the patients. There might have been an influence of the rheological disturbance in these vessels on ON. Seventh, Image J was used in this study because RTVue XR Avanti and Cirrus HD-OCT 5000 did not measure the vessel density. However, Image J is used throughout the world as standard software to evaluate images.

In conclusion, decreased retinal perfusions in eyes with good visual acuity after treatment for ON were shown noninvasively by OCT angiography. The retinal thicknesses also decreased in all eyes. ON may cause not only retinal structural damage, but also a decreased retinal perfusion, even when visual acuity recovered well after treatment.

## Supporting information

S1 Table Specific dataset for all individuals. (XLSX)

S2 Table STROBE_checklist. (DOC)
